# Recommendations for the organization of the teleconsultation service in a telestroke network

**DOI:** 10.1186/s42466-024-00318-3

**Published:** 2024-04-25

**Authors:** Hanni Wiestler, Philipp Zickler, Hebun Erdur, Mazen Abu-Mugheisib, Bernd Kallmünzer, Caroline Klingner, Peter Müller-Barna, Gordian Hubert, Christoph Gumbinger, Hans Worthmann

**Affiliations:** 1 Department of Neurology, Academic Teaching hospital of the Ludwig-Maximilians-University, München Klinik, Munich, Germany; 2 Department of Neurology and Clinical Neurophysiology, University Augsburg, Augsburg, Germany; 3 Department of Neurology, Asklepios Fachklinikum Teupitz, Teupitz, Germany; 4 Department of Neurology, Municipal Hospital Braunschweig, Braunschweig, Germany; 5 Department of Neurology, Universitätsklinikum Erlangen, Friedrich-Alexander-Universität Erlangen- Nürnberg, Erlangen, Germany; 6 Department of Neurology, Jena University Hospital, Jena, Germany; 7 Department of Neurology, University Heidelberg, Heidelberg, Germany; 8 Department of Neurology, Hannover Medical School, Hannover, Germany; 9 Department of Neurology, Charité University Hospital, Berlin, Germany

**Keywords:** Telestroke network, Teleconsultation service, Acute stroke, Telemedicine, Recommendations

## Abstract

Telestroke networks aim to improve acute stroke care within their catchment area. Through a teleconsultation service, the network centers provide support to network hospitals that lack continuous neurological expertise for acute stroke management decisions. Although the importance of telemedical treatment in stroke care is steadily increasing, so far no standards exist for the organization of the teleconsultation service in networks.

To ensure a high-level of quality for all processes and measures concerning telemedical stroke treatment, the commission for telemedical stroke care of the German Stroke Society (Deutsche Schlaganfall-Gesellschaft, DSG) created the following recommendations on how to organize a teleconsultation service within a telestroke network. The recommendations are the result of an adjustment process between the authors and include guidance on requirements, qualifications, processes and quality management within the teleconsultation service.

## Background

Telemedical consultation is a valuable treatment option for acute ischemic stroke in hospitals without around-the-clock stroke expertise on-site. It has been evaluated in different telestroke network settings worldwide and was shown to be safe and reliable [1–5]. More and more telestroke networks are being established, constantly improving stroke care particularly in rural areas [6–8]. This includes the improved access to evidence-based therapies like intravenous thrombolysis (IVT) and mechanical thrombectomy (MTE), as well as access to specialized acute stroke care by implementation of tele-stroke units. While a prior publication of the Telestroke Committee of the European Stroke Organisation contains recommendations and requirements for the implementation of a stroke network in general [9], there are still no published standards or recommendations focusing on how to organize the teleconsultation service within a telestroke network in detail.

## Methods

These recommendations were developed and consented on behalf of the commission for telemedical stroke care of the German Stroke Society (Deutsche Schlaganfall-Gesellschaft, DSG) and are based on the expert consensus of the authors and other members of the commission and related literature. All authors are experienced stroke neurologists with long-term experience in telemedical stroke care. They represent eight different German stroke networks of varying network sizes, numbers of yearly consultations and financing models, making these recommendations broadly applicable. The recommendations were originally developed in German language, then translated and slightly shortened for publication.

## Main text

### Scope, principles, and terminology

The present recommendations are directed towards telemedical stroke networks and are tailored to the diagnosis and treatment of neurovascular diseases, thereby limiting their unrestricted application to the telemedical management of other neurological conditions. They delineate recommendations for the organization of a teleconsultation service, provided by tertiary stroke centers, to cooperating hospitals within a stroke network. This includes guidance on qualifications, processes and workflow standards within the teleconsultation service (e.g., indications, execution, documentation standards). Established certification criteria for stroke units and (tele)stroke networks are referred to but not described in detail in this work [10–12]. 

The aim of a telestroke network is to improve acute stroke care within the region covered by the network. An essential component of their work is the teleconsultation service, which continuously offers neurovascular expertise for acute diagnostic and therapeutic decisions and utilizes existing network structures to ensure a timely and best possible acute stroke management across hospitals of different levels of care (e.g., by organizing transfers for MTE or neurosurgical interventions). The teleconsultation service within a telestroke network acts as an advising institution for all neurovascular questions and their differential diagnosis. Conducting a teleconsultation does not establish an independent contractual relationship between the telestroke centers and the treated patients. The participating physicians in the cooperating hospitals are usually not employed nor engaged in an employment-like relationship with the network centers. The same applies in reverse for the telestroke physicians of the network centers with respect to the cooperating hospitals.

A teleconsultation is defined as the process starting with the initial contact made by the cooperating hospital with the network center and concludes with the recommendations from the telestroke physician and the creation of a medical report. A teleconsultation typically comprises the following individual elements: initial patient presentation, video conference, co-assessment of imaging data and establishing a diagnosis, resulting in recommendations for the further management, diagnostic and/or therapeutic measures. Usually, a teleconsultation process includes all the aforementioned individual elements, which may require repeated contacts. As needed, only specific individual elements may be conducted. Every teleconsultation should be documented in a medical report, which is subsequently transmitted to the treating hospital.

### Requirements for the teleconsultation service

#### a. Basic and organizational requirements

The teleconsultation service is available 24/7/365 and immediately accessible for stroke-related inquiries within an immediate response time. An outage concept in case of temporary disruption of telephone accessibility (e.g., due to technical disturbances) needs to be established. The telestroke physician is released from other time-critical activities while on duty. A sufficient number of telestroke physicians is required to ensure an around-the-clock teleconsultation service and management of additional administrative tasks (see below). The number of physicians required depends on the size of the network and the number of consultations. The treatment of neurovascular emergencies is extremely time-critical, which is why the principle of “time is brain” must be taken into account at all times, especially for patients potentially undergoing revascularization therapy.

#### b. Qualification requirements for telestroke physicians

All physicians performing the teleconsultation service are board-certified neurologists or, alternatively, residents in neurology with at least 4 years of training in neurology, including a minimum of 1-year experience in a stroke unit or intensive care unit. For standardized training of new staff, we recommend the definition of a training concept that addresses the special features of the telemedical treatment, including potential limitations and uncertainties compared to conventional face-to-face neurological examination.

#### c. Workplace requirements

Both visual and acoustic data security must be ensured when using the teleconsultation workstations. Workstations used must generally be secured against unauthorized access. Consequently, using a mobile or external workstation in home-office is generally only permissible in a private environment. An interruption-free and data protection-compliant environment must be ensured. Unauthorized listening by third parties should be prevented by using headsets and/or keeping doors closed. Passing on access data for the use of the service programs is prohibited and the storage of patient data on a mobile workstation (laptop) is generally not permitted. The video consultation is carried out bidirectionally, i.e. the telestroke physician on duty should be visible to the staff in the cooperating hospital and to the patient.

#### d. Technical requirements

All technical solutions must comply with the data protection and Information Technology (IT) security regulations valid within the region of the telestroke network, considering the special requirements for healthcare purposes and critical infrastructures. For this, the technical solution has to be checked and approved by responsible IT and data security professionals.

Providing teleconsultation services in a private setting is generally feasible if meeting the requirements outlined in 2.c. A sufficiently fast Internet connection is necessary for interruption-free data transmission. Using a sufficiently secured private WLAN or LAN internet connection is permitted. Alternatively, the use of data transmission via a mobile internet module (e.g. LTE module) is possible as long as sufficient reception is guaranteed. However, data transmission must be secured in accordance with standards mentioned above. Each workstation should be equipped with a high-performance mobile Internet module serving as a backup solution in case of local internet connection failure.

Before starting the teleconsultation service, we recommend to ensure the functionality of the system, including sufficient data transfer rates for stable and high-quality audio and video transmission. To guarantee high reliability, the functionality of the private internet connection should be verified before starting services outside the clinic. In addition, at the beginning of each shift or after any change on the phone settings (e.g., call forwarding), a test call should be carried out to confirm the telephone accessibility through the service number. Continuous telephone accessibility during the service is indispensable. The telestroke physician needs to be familiar with the technical equipment and its functionalities, as well as problem solving strategies in the event of a malfunction.

### Indications for starting a teleconsultation

In general, whenever the medical staff at the cooperating hospital suspects or diagnoses an acute cerebrovascular disease in a patient and there is no neurologist immediately available on-site, an indication for teleconsultation emerges. Also, when neurological deficits appear unclear or uncertain, a teleconsultation is sensible to discern rare stroke manifestations and stroke mimics. Whether the suspicion is justified and further telemedical measures are necessary, is determined by the professional assessment of the telestroke physician, the capabilities of the cooperating hospital and the contractual agreements within the network. Other neurological and non-neurological diseases that do not fall within the field of vascular neurology do not constitute an indication for a telestroke consultation, if a cerebrovascular diagnosis can be securely excluded already at this time. In individual cases of time-critical neurological emergencies (such as status epilepticus, herpes encephalitis, among others), involving neurological expertise via teleconsultation may be indicated if an alternative neurological consultation on-site or secondary transport is associated with a time delay that may be associated with a risk for the patient. In general, the treatment of non-vascular issues is not the primary goal of a telestroke network. However, networks are free to provide their teleconsultation service to cooperating hospitals for other (acute) neurological queries, as long as the acute treatment of stroke patients is not delayed or disadvantaged as a result.

#### a. Acute cerebrovascular issues

If, based on the reported symptoms upon announcement of the consultation, there is a suspicion of an acute stroke (or an underlying stroke cannot be ruled out), with symptom onset within the last 72 h or worsening or fluctuation of symptoms within this timeframe, a teleconsultation is clearly indicated. This includes both ischemic and hemorrhagic strokes as well as transient ischemic attacks (TIA). In certain cases, a teleconsultation may also be indicated at symptom onset > 72 h (e.g., high-risk patients with recurrent TIA, unclear onset and last seen well > 72 h, “warning hemorrhage” in subarachnoid hemorrhage with evidence of an aneurysm, etc.). It is at the discretion of the telestroke physician to assess whether a teleconsultation is indicated and which components it should include.

If there is suspicion of another acute cerebrovascular condition requiring further diagnostics or treatment (e.g., cerebral venous and sinus thrombosis, retinal central artery occlusion, etc.), a teleconsultation is also indicated.

There is no general indication for a teleconsultation in the case of trauma-related condition, including traumatic intracranial hemorrhages (with few exceptions, e.g., ischemic infarction as a result of a traumatic dissection). However, referral to the nearest suitable neurosurgical clinic should take place and be documented. If a neurologist is present on-site, acute stroke treatment can be provided in whole or in part on-site (with or without additionally involving the teleconsultation service).

#### b. Acute thrombectomy queries

If a cerebral large vessel occlusion requiring intervention is suspected or diagnosed, a telemedical consultation should be realized for specialized assessment, indication for intervention and support in organizing further care (e.g. transfer to a thrombectomy center, initiation of a MTE on-site by a mobile intervention team) - even if a neurologist or specialist is present at the cooperating hospital. A video examination is usually not necessary when neurological expertise is available on-site while transfer and co-assessment of imaging through the teleconsultation service is still recommended. In order to fulfill this function according to the latest treatment criteria, the telemedical service needs to be associated with a neurovascular tertiary care center including a certified stroke unit and 24/7/365 neurointerventional service.

#### c. Teleconsultations for complex cerebrovascular issues

With its neurovascular expertise, the teleconsultation service is also available to advise on-site neurologists on complex cerebrovascular issues, regardless of whether they relate to acute treatment decisions. In the case of competing requests, acute treatment queries are to be prioritized to consultations of mid or low acuity.

#### d. (Semi-)elective cerebrovascular queries as an additional service

In the case of elective cerebrovascular issues, a teleconsultation can be offered as an additional service to the cooperating hospitals if the teleconsultation service has capacity for it, especially if there are therapeutic implications. In any case, this must not delay the processing of acute consultations.

### Procedure of a teleconsultation

#### a. Overview

**Fig. 1:**
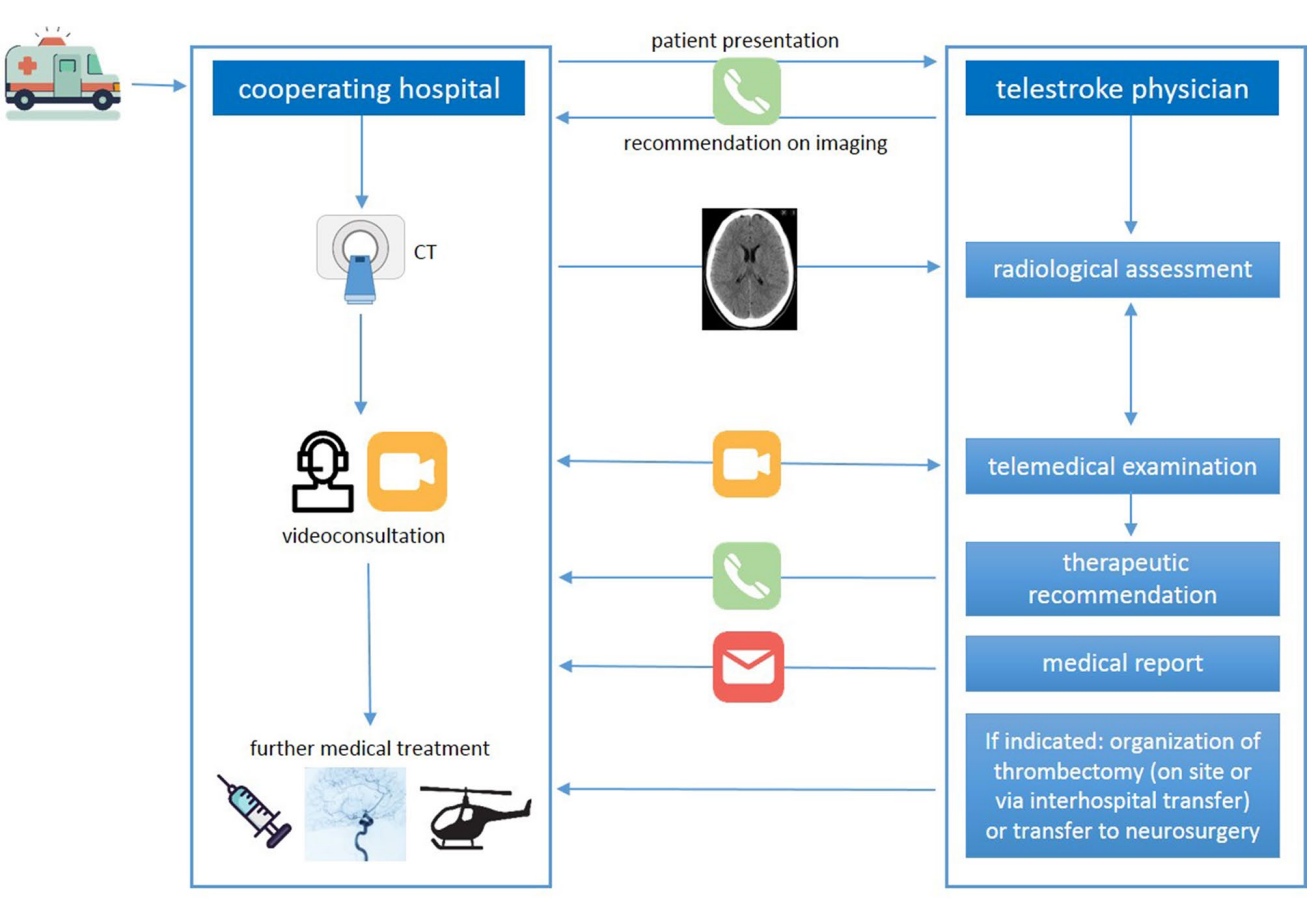
Schematic representation of the main tasks within a teleconsultation

The teleconsultation begins with the first call from the cooperating hospital to the teleconsultation service for the presentation of a patient. From this point onwards, parallel processes take place on both sides (cooperating hospital and telestroke physician) as part of the acute treatment, which are shown in Fig. 1; Table 1. Depending on the specifics of the network, there may be deviations regarding the individual steps. Most important is that an internal network procedure is defined with the aim of reaching a decision and initiating treatment as quickly as possible. Depending on the case, individual components may be omitted. A second and third telephone call are usually only necessary if a treatment recommendation is not directly given during the video consultation.

**Table?1:**
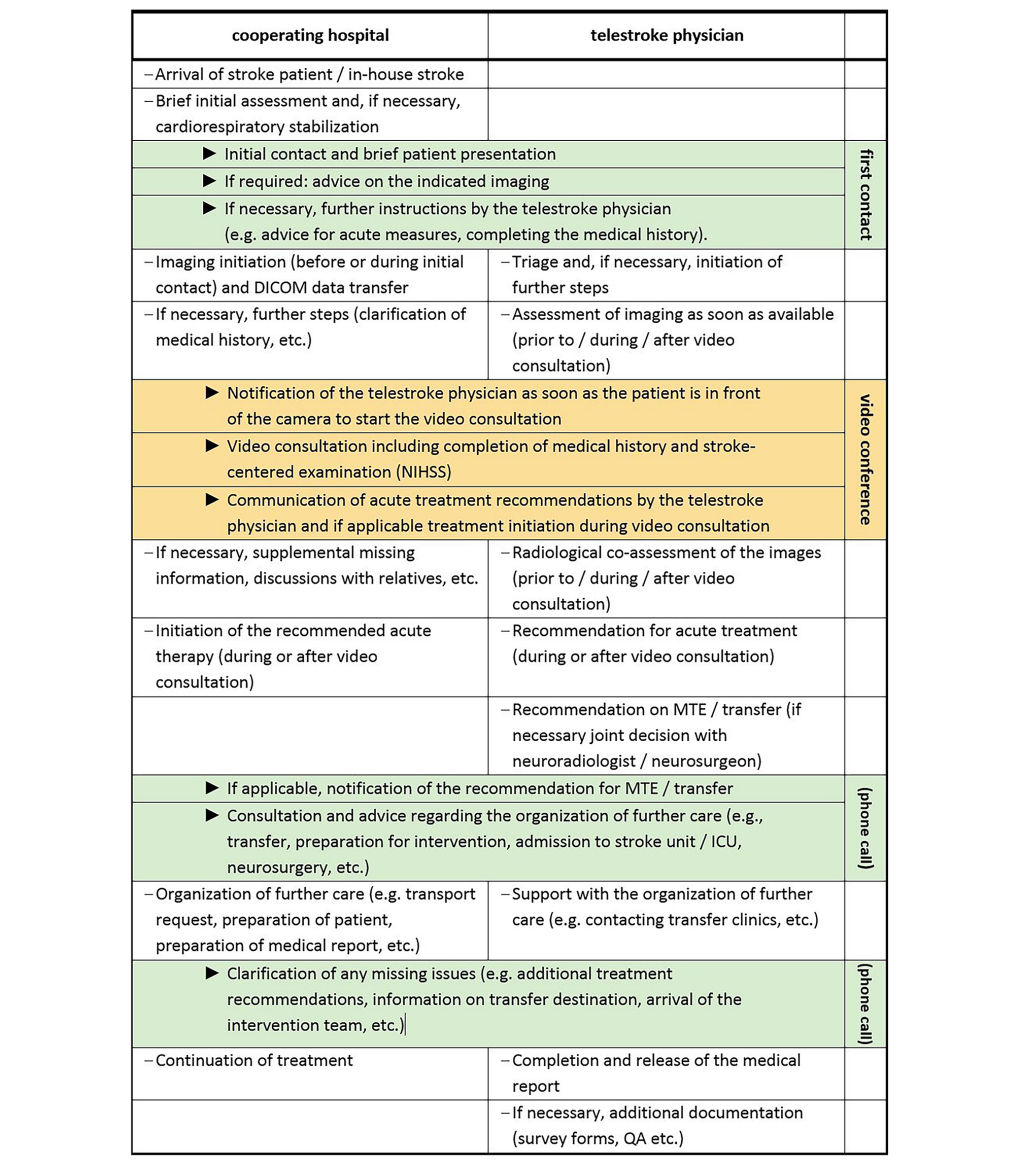
Scheme of the teleconsultation procedure

Direct contacts between the cooperating hospital and the telestroke physician are marked with ► (in yellow: processes reg. video conference; in green: further contacts, e.g. by phone)

#### b. Initial contact / patient registration

When a patient with a suspected stroke arrives, the physician on-site should inform the teleconsultation service immediately after an initial brief assessment, but at the latest while the imaging is being carried out. Patient presentation is usually done by telephone so that any uncertainties can be clarified from the outset. Alternative registration systems are conceivable (e.g., virtual documentation platform, smartphone applications, etc.), provided that the telestroke physician receives all triage-relevant information; in the event of any remaining open questions, those can be clarified through an additional phone call anyway.

The following information should be communicated during the initial phone call: brief and concise description of the current clinical syndrome, important associated symptoms and circumstances (e.g., existing contraindications for IVT), patient’s prior condition (e.g., severe dependency on care or dementia, bedridden status, etc.), and information related to the time window (onset, if known; otherwise, last seen well and time of discovery). In addition, relevant previous illnesses and relevant previous medication can already be requested here. If relevant information is missing, the telestroke physician instructs the medical staff on-site to obtain it while the imaging is being performed. If there is a need for advice regarding the indicated imaging, the teleconsultation service will make an imaging recommendation after obtaining the relevant information. The indications for the various imaging modalities should be regulated within the network and take local resources into account.

After the initial contact, the indicated imaging is carried out as quickly as possible and then immediately transmitted to the telestroke physician. In the meantime, the patient is brought to the teleconsultation room as quickly as possible. As soon as the video consultation is ready to be started the telestroke physician has to be informed.

For quality assurance purposes it is recommended to record the time of the initial contact.

#### c. Video consultation

During video consultation, the telestroke physician briefly confirms the patients’ medical history in a short survey and asks for any missing information. For that, a standardized checklist should be used to acquire all necessary information in a structured manner (e.g., potential contraindications to IVT). Subsequently a focused and symptom-oriented neurological examination is carried out by the telestroke physician with support of the on-site physician with the focus on NIHSS assessment, but also to distinguish differential diagnoses where necessary. If all necessary information is already available during the video consultation (including co-assessment of the imaging), a therapeutic recommendation can already be made; in addition, if applicable, the telestroke physician informs the patient about individual indication and potential risks, especially in case of off-label therapy. The time of the video consultation is recorded for quality assurance purposes. A video examination can generally be omitted when a neurologist is available on-site.

#### d. Imaging assessment

As soon as available, the teleconsultant evaluates the imaging as this will be needed for any further treatment decisions. The telestroke physician must be able to call a (neuro-)radiologist at any time for co-assessment of the imaging and expert advice. In more detail, this may vary from case to case and may involve consultation with the radiologists in the cooperation hospital or, especially in case of decisions involving MTE, responsible (neuro-)radiologists in neurovascular centers within the telestroke network. The specific responsibilities, e.g. type and contractual details of cooperation with (neuro-)radiology, should be regulated within the network. If necessary, additional imaging can be recommended. The times of imaging performance and imaging transmission are recorded for quality assurance purposes.

#### e. Recommendations on acute recanalization therapies and early stroke management

Based on the available findings, the telestroke physician establishes a diagnosis and provides a recommendation on acute stroke treatment, which involves the decision for or against acute recanalization therapies and general recommendations regarding acute stroke management and early secondary prevention. If a neurologist is present in the cooperating hospital, the decision for initiating MTE is generally made by or in agreement with the on-site neurologist, whereas IVT and early stroke management is typically entirely managed by the on-site neurologist. Furthermore, the decision on MTE should always involve the radiological expertise by the neuroradiologist who should perform the intervention.

In principle, it is the responsibility of the local medical staff to check for potential contraindications for IVT (or any other administered therapy), e.g. using a checklist provided by the network center. Nevertheless, it is recommended that the telestroke physician obtains confirmation from the local medical staff that the check has actually been made and that there are no contraindications before recommending IVT (or MTE). If there are any uncertainties, questionable or relative contraindications a risk-benefit assessment needs to be made jointly supported by the telestroke physician. The telestroke physician also supports the on-site medical staff in providing treatment information to the patient. However, it remains the responsibility of the medical staff at the cooperating hospital to ensure that the patient has been sufficiently informed about the treatment and that verbal and/or written consent has been obtained before initiating therapy. In the case of patients who are incapable of giving consent, consent can be assessed based on the patient’s presumed or previously expressed wishes. Adequate documentation of the treatment decision is necessary, especially in borderline decisions (pro or contra) and off-label therapies.

Following the decision to proceed with IVT, the staff on-site is instructed to initiate bolus administration immediately, preferably during or directly after the video conference. In case of uncertainties, the telestroke physician provides assistance to the local staff via video conference or by telephone (e.g. dose determination, application method, etc.). When organizing MTE, the telestroke physician supports the team on-site or preferably even takes on a leading role in organization (e.g., contacting thrombectomy centers, initiating MTE through a mobile intervention team). However, the primary responsibility remains in the on-site hospital and it can be conflicting that a high volume of time-critical teleconsultations reduces time capacity of the telestroke physician so that transfer organization for MTE might be assigned more to the on-site medical staff as required. Network-specific agreements deviating from this approach are possible, provided that standards for transfer organization exist, and quality assurance measures ensure well-working transfer processes without any avoidable loss of time.

Network-specific standards concerning the processes related to IVT and MTE (e.g., decision-making, responsibilities, procedures, post-treatment), but also regarding basic stroke management and secondary prophylactic treatments should be provided by the network coordination. These contents are made available to the involved staff in written form and additionally communicated as part of regular trainings. To ensure the fastest possible treatment initiation for time-critical therapies as MTE, networks are encouraged to define standards for a structured interhospital transfer management. These standards regulate indications and processes regarding interhospital patient transfer (also beyond MTE) in consent with network partners and neurovascular centers within their service area and also take into account the establishment and maintenance of digital image forwarding. Process times should be recorded for quality assurance purposes.

#### f. Documentation guidelines

Even if the telestroke physician does not confirm the suspected diagnosis of stroke after initial patient presentation and no video examination is carried out, it is necessary to create a medical report that logically and comprehensibly states the assessment. Any kind of medical advice should be recorded in a consultation report, including potentially recommendations on non-vascular issues (e.g. anti-epileptic therapy, etc.). The same applies to any co-assessments of imaging directly involving the teleconsultation service.

A complete consultation report contains information on the patient’s medical history, the neurological examination findings including the complete NIHSS (if a video consultation was carried out in the absence of a neurologist on-site), results of imaging assessment, the (suspected) diagnosis (including etiology, localization and type of vascular pathology), as well as diagnostic and therapeutic recommendations. Particularly in the case of borderline decisions and treatments beyond standard approval criteria, the treatment recommendations should include a plausible and comprehensible reasoning and documentation of the decision-making process. If the teleconsultant was involved in patient information and obtaining informed consent for acute treatment options this should also be documented. It is also recommendable, to record any third parties involved in the decision-making process (e.g. involved radiologists, supervising senior physicians) including name and function.

### Personnel resources and task description

The basic personnel requirements for operating a teleconsulting service, as well as required professional qualification for telestroke physicians are described earlier. For administrative tasks, supervision and general coordination, the teleconsulting service is led by a senior physician.

The tasks described below mainly relate to the services within the teleconsultation service. In addition, there may be responsibilities in other areas within the network administration. The responsibilities should be regulated within each network depending on the individual structural and personnel requirements.

The tasks of the teleconsultant neurologists include.

– Round-the-clock teleconsultation service for all cooperating hospitals (24/7/365).
– Supporting interhospital patient transfers for further care according to indication.
– Documentation of each teleconsultation performed by creating a medical report.
– Recording of quality assurance-relevant data points (e.g., process times in acute treatment).
– Handovers between shift changes.
– Registration of problems within the teleconsultation service (e.g. procedural failures, communication problems) and feedback to the teleconsultation service/network management.
– Feedback and solution of technical problems (if necessary in cooperation with technical support).
– Participation in the induction of new employees.
– Participation in regular team meetings (case discussions, problem-solving sessions, internal team training).
– Attendance at training sessions; if applicable, organization of training sessions, workshops, bedside visits on-site, creating and updating training materials.
– Support in reviewing and updating workflows, recommendations and decision-making aids for diagnostics and therapy within the teleconsultation service and the stroke network in general.
– Depending on the network and personnel structure, additional administrative activities can also be transferred to the telestroke physicians (e.g., support of cooperating hospitals, conducting clinic visits, involvement in scientific or quality assurance projects).

The tasks of the senior physician responsible for the teleconsultation service include:

– Creation, reviewing and updating of treatment recommendations and decision-making aids for diagnostics and therapy, as well as Standard Operating Procedures (SOPs) to regulate organizational processes within the teleconsultation service and the stroke network in general.
– Development and implementation of a quality assurance concept, as well as monitoring and optimization of structural and procedural quality in the teleconsultation service.
– Development and implementation of an interhospital transfer management concept for optimizing patient transfers within the network’s service area, considering pre-hospital processes and conditions, and in coordination with the neurovascular centers providing further care (e.g., MTE).
– Organization and leadership of regular team meetings (case discussions, problem-solving sessions, internal team training).
– Responsibility for the induction of new employees.
– Point of contact for issues within the teleconsultation service and processing of feedback between the teleconsultation service and cooperating hospitals.
– Point of contact for organizational, medical, and technical queries from the teleconsultation team (e.g., in the form of a background service).
– Responsibility for service schedules, duty rosters, holiday regulations, and personnel management within the teleconsultation service.
– Responsibility for the function and optimization of the technical teleconsultation environment including digital image transfers within the network.
– Development, implementation, and/or management of additional network-internal and network-crossing scientific or quality assurance projects.
– Exchange and communication with other stroke networks and participation in cross-network projects, as well as collaboration with institutions of national and international professional societies to improve (telemedical) stroke care.
– Representation of the network.

### Quality Management within the teleconsultation service

To ensure a high standard of treatment quality provided by the teleconsultation service, it is necessary to define network-specific concepts for quality assurance. Besides providing optimal treatment according to the current standards of evidence-based medicine, focusing on rapid treatment initiation is crucial, requiring specific personnel, technical resources, and standardized workflows. The goal of a quality management concept lies not only in defining quality standards but also in quality control and improvement according to the PDCA cycle (Plan, Do, Check, Act).

The quality management concept of a telestroke network goes far beyond quality assurance in the teleconsultation service and includes quality control measures in cooperating hospitals. These recommendations focus on quality assurance measures directly associated with teleconsultation service procedures.

The following chapters provide an overview of possible quality assurance measures in the teleconsultation service without claiming to be exhaustive.

#### a. Quality planning and implementation (“Plan”, “Do”)

Quality planning is primarily based on defining quality standards, with staff training being central to its implementation alongside resource provision. Measures for quality planning and implementation in the teleconsultation service include:

– Defining personnel, technical and workspace requirements.
– Establishing and applying network-specific SOPs for organizational workflows, structures, and processes.
– Providing and regularly updating treatment recommendations for the teleconsultation team.
– Developing a service schedule adapted to the volume of consultations and considering specifics in telemedical treatment.
– Creating a structured induction concept for new teleconsultation service staff.
– Organizing regular trainings and team meetings for the teleconsultation team.
– Providing suitable working materials to facilitate processes in the teleconsultation service (e.g., contact/phone lists, checklists, medical history forms, etc.).
– Offering regular training, bedside teachings and continuing education of stroke care-involved personnel in cooperating hospitals (only indirectly related to, but essential for teleconsultation service processes).

#### b. Quality Control (“Check”)

Once the standards have been defined, control mechanisms should be introduced. They primarily serve to identify systematic and procedural problems so that appropriate measures can be introduced to maintain or improve quality.

– *Capturing and evaluating defined measurable quality indicators* (e.g., response time, process times, therapy rates etc.). For this working with already existing quality criteria of related professional societies or established quality control authorities is recommended. In addition network- and teleconsultation-specific criteria can be defined. The aim of these evaluations is primarily to detect systematic procedural problems and disruptive factors in order to be able to counteract these with suitable measures. In addition, trends can be identified from which conclusions can be drawn about necessary or successfully implemented measures.
– *Standardized review of teleconsultations*: this tool is particularly suitable for assessing the content quality of teleconsultation service. A predefined criterion catalogue is recommended to standardly review the content quality of teleconsultations. Deviations from the standards can thus be reported directly to the teleconsultants concerned and discussed. Regular evaluations of the overall data can indicate systematic difficulties or highlight trends.
– *Feedback system*: Using a standardized demanding feedback system, difficulties within the teleconsultation service can be promptly identified and addressed. In addition to immediate insights gained through feedback processing, a standardized evaluation of the collected data can be conducted to identify issues and trends in a manner similar to the aforementioned instruments.
– *Audits*: Conducting external audits within the network centers, encompassing the processes of the teleconsultation service, along with audits for cooperating hospitals, represents additional potent measures for quality control.

#### c. Quality Improvement (“Act”)

Measures to continuously improve the quality of the teleconsultation service are derived from the knowledge gained through quality control.

Examples of quality improvement measures include:

– Communication and processing of systematic problems or critical individual cases with involved individuals or groups.
– Problem-specific individual or team training.
– (Interdisciplinary) case presentations and discussions.
– Review and, if necessary, adjustment of established processes and procedures.
– If necessary, extending existing SOPs and treatment recommendations or creation of new standards or decisions aids.
– Optimization of working conditions (working environment, technology, equipment, etc.).

## Conclusions

The implementation of a teleconsultation service within a telestroke network requires several considerations and a high degree of standardization and organizational work to secure best possible telemedical stroke treatment. Aside from fulfilling certain basic, personnel and technical requirements, defining procedural standards helps to ensure a consistent level of quality, auditability and transparency within the telestroke network that should be equivalent to a non-telemedical stroke management concept. The here described recommendations address both existing and future telestroke networks, providing guidance on how to organize a teleconsultation service.

## Data Availability

Not applicable.

## Abbreviations

DSG: Deutsche Schlaganfall‐Gesellschaft
NIHSS: National Institutes of Health Stroke Scale
PDCA: Plan, Do, Check, Act
QA: Quality Assurance
SOP: Standard Operating Procedure
TIA: Transient Ischemic Attack
IVT: Intravenous Thrombolysis
MTE: Mechanical Thrombectomy
WLAN: Wireless Local Area Network
LTE: Long Term Evolution
DICOM: Digital Imaging and Communications in Medicine
IT: Information Technology
